# Preserving brain function in a comatose patient with septic hyperpyrexia (41.6 °C): a case report

**DOI:** 10.1186/s13256-017-1204-8

**Published:** 2017-02-13

**Authors:** Samantha Sterkel, Akinboyede Akinyemi, Marcos A. Sanchez-Gonzalez, George Michel

**Affiliations:** 1 Department of Internal Medicine, Larkin Community Hospital, Graduate Medical Education, 7000 SW 62nd Avenue, Suite 401, South Miami, FL 33142 USA; 2 Department of Psychiatry, Larkin Community Hospital, Graduate Medical Education, 7000 SW 62nd Avenue, Suite 401, South Miami, FL 33142 USA; 3 Division of Clinical & Translational Research, Larkin Community Hospital, South Miami, FL USA

**Keywords:** Hyperpyrexia, Levetiracetam, Glutamate, Organum vasculosum laminae terminalis, Neuroprotection, Amantadine, Glasgow Coma Score

## Abstract

**Background:**

Pyrexia is a physiological response through which the immune system responds to infectious processes. Hyperpyrexia is known to be neurodegenerative leading to brain damage. Some of the neurotoxic effects of hyperpyrexia on the brain include seizures, decreased cognitive speed, mental status changes, coma, and even death. In the clinical management of hyperpyrexia, the goal is to treat the underlying cause of elevated temperature and prevent end organ damage.

**Case presentation:**

This case illustrates a 39-year-old white American man referred from another medical facility where he had undergone an upper gastrointestinal tract diagnostic procedure which became complicated by blood aspiration and respiratory distress. During hospitalization, he developed a core body temperature of 41.6 °C (106.9 °F) leading to cognitive decline and coma with a Glasgow Coma Score of 3. Levetiracetam and amantadine were utilized effectively for preserving and restoring neurocognitive function. Prior studies have shown that glutamate levels can increase during an infectious process. Glutamate is an excitatory neurotransmitter that is utilized by the organum vasculosum laminae terminalis through the neuronal excitatory system and causes an increase in body temperature which can lead to hyperpyrexia. Similar to neurogenic fevers, hyperpyrexia can lead to neurological decline and irreversible cognitive dysfunction. Inhibition of the glutamate aids a decrease in excitatory states, and improves the brain’s regulatory mechanism, including temperature control. To further improve cognitive function, dopamine levels were increased with a dopamine agonist.

**Conclusions:**

We propose that a combination of levetiracetam and amantadine may provide neuroprotective and neurorestorative properties when administered during a period of hyperpyrexia accompanied by any form of mental status changes, particularly if there is a decline in Glasgow Coma Score.

## Background

Fever is a natural regulatory protective mechanism through which the human body counteracts physiological insults. Causes of fever include infections, traumatic brain injury, thalamic injury, dehydration, tissue destruction, and thromboembolism [1, 2]. Conversely, hyperpyrexia or body temperature greater than or equal to 41.5 °C (106.7 °F) may lead to pathological complications including detrimental mental status changes, neuronal brain injury, cognitive decline, coma, and even death [2]. Causes of hyperpyrexia include neuroleptic malignant syndrome, heat stroke, amphetamine overdose, serotonin syndrome, infections, and sepsis [2]. In a study conducted by Minamisawa *et al*. [3], it was shown that hyperpyrexia induced pannecrosis as well as damage to the neocortex, caudoputamen, and substantia nigra pars reticulate, which are responsible for dopamine activity. These areas of the brain are involved in conscious reasoning, spatial orientation, sensation to all modalities, movement regulation, and learning [3]. Regulation of neuronal excitability occurs in the brain via balancing of gamma-aminobutyric acid (GABA) and glutamate decarboxylase [4, 5]. Glutamate is a major excitatory neurotransmitter in the brain and serves as a precursor for the synthesis of GABA, which can be inversely metabolized back to glutamate via the tricarboxylic acid (TCA) cycle [6]. Dysfunction of neuronal activities in hypoxia, however, causes poor regulation of TCA cycle causing uncontrolled release of glutamate which becomes toxic to neurons and thus inducing further neuronal necrosis in the brain [7].

Hyperpyrexia of infectious origin is discussed in this case in combination with the pharmacological interventions utilized following a severe mental status decline leading to a comatose state. A combination of levetiracetam and amantadine were successfully utilized. Here, we demonstrate the beneficial effect of neuroprotective agents in fever regulation via the organum vasculosum laminae terminalis (OVLT) and its function in fever regulation in a patient with brain injury resulting from hyperpyrexia. The OVLT glutamate spike has been studied in animals showing a rise in glutamate levels prior to measurable increase in body temperature [8]. This suggests a link between hyperpyrexia and OVLT involvement in temperature regulation via glutamate release [9]. While levetiracetam is a known antiepileptic agent, its impact on regulating the integrity of the blood–brain barrier function following hyperpyrexia in a comatose state has not been well documented in humans [10]. Levetiracetam is documented with a known mechanism of action in neuronal glutamate regulation in seizure disorder via inhibition of presynaptic calcium channels [11]. Following the assessment and review of this case, we suggest that in patients with hyperpyrexia and cognitive decline, a combination of amantadine and levetiracetam could be beneficial for preserving neuronal survival and restoring cognitive function.

## Case presentation

A 39-year-old white American man with a past medical history of hypertension, hyperlipidemia, and major depressive disorder presented to our facility after being transferred from another facility following an attempted suicide. He presented to our intensive care unit; he was intubated due to respiratory distress and hypoxic-anoxic brain injury, secondary to complications of sharp foreign object ingestion as a means of suicide attempt. According to collateral information retrieved from the sending facility, he was seen aspirating blood while undergoing esophagogastroduodenoscopy (EGD) thus requiring emergency intubation. On admission to our facility, an abdominal X-ray showed no evidence of foreign object ingestion. Another EGD was performed at our facility to investigate upper gastric bleeding which revealed a 2 cm ulcer at the base of his esophagus, which was cauterized resulting in control of the hemorrhage. An abdominal axial computed tomography (CT) scan performed on hospital day 18 showed evidence of a foreign object in his descending colon which was later expulsed via bowel movement on day 20. Per transfer records received at our facility, medications included amlodipine 5 mg orally daily, simvastatin 10 mg orally daily, gemfibrozil 600 mg orally twice a day, and niacin 500 mg daily. According to the history obtained from members of his family, there were no reported medication allergies and no past non-prescribed substance use history.

Within hours of admission to our intensive care unit, his mental status progressively declined due to hypoxic-anoxic brain injury reaching a Glasgow Coma Score (GCS) of 6. On physical examination there was no eye opening response, no verbal response, and he only merely withdrew from painful stimuli. There was neither decorticate nor decerebrate posturing at the time of examination. Lung auscultation revealed decreased breath sounds in bilateral lung bases. His abdomen was distended, obese, with decreased bowel sounds in all four quadrants and tympanic to percussion. His white blood cell (WBC) count was elevated at 14.9 (10^3^/uL), with 83.6 % neutrophil predominance. A chest X-ray showed bilateral pleural effusion. A sputum culture was positive for *Klebsiella pneumonia.* His clinical picture was consistent with aspiration pneumonitis with severe hypoxia which later decompensated to ventilator-associated pneumonia with methicillin-resistant *Staphylococcus aureus* (MRSA) pneumonia (as seen on sputum culture), which thus made him ventilator dependent due to decreased respiratory drive. His treatment was a combination of levofloxacin 750 mg administered intravenously daily, piperacillin/tazobactam 3.375 mg every 6 hours, and vancomycin 1 gram daily. On day 8, a maximum core temperature (T_max_) of 39.6 °C (103.3 °F) was recorded rectally. He had no motor response to painful stimuli, no eye opening, nor verbal response despite being off all sedating agents. His blood culture at this time was positive for *Candida albicans* and, as a result, micafungin 100 mg administered intravenously daily was initiated. While on this regimen, on day 9, a temperature of 41.6 °C (106.9 °F) was recorded with a GCS of 3. Acetaminophen 650 mg was administered rectally; cooling blankets and multiple ice packs were applied to his groin, axillae, and neck regions resulting in a decrease in his body temperature to 37.8 °C (100.1 °F) within 8 hours of intervention. He continued to have fever on a daily basis with no improvement in cognitive functioning as he remained comatose. On hospital day 11, he had become flaccid and was considered to be in a vegetative state as evidenced by an electroencephalogram (EEG) study, which showed diffuse slowing consistent with encephalopathy with very poor prognosis. His WBC count increased to 27.9 (10^3^/uL) and he remained in a state of septicemia. Due to multiple failed attempts to wean him off the ventilator, a tracheostomy was performed on day 14 and a percutaneous endoscopic gastrostomy (PEG) tube was placed on day 17. Given his neurological decline, a lumbar puncture was considered for cerebrospinal fluid (CSF) analysis. However, he was already on broad spectrum antibiotics, therefore, the benefits from CSF analysis would have been undermined by antibiotic coverage.

After concerted deliberations over the trial of levetiracetam as a neuroprotective and antiepileptic agent, the decision was made on day 29 to administer 500 mg of the medication via a PEG tube at bedtime. Within 48 hours of administration, he was awake and alert but remained disoriented to person, place, and time. His GCS improved from 3 to 14 and he was able to respond to painful stimuli from his lower extremities up to his knees. Amantadine 50 mg/ml via a PEG tube every morning was started for neurocognitive stimulation as he was noted to have decreased cognitive speed measured grossly during conversation evidenced by increased latency of response when questioned. On hospital day 38 (9 days after levetiracetam 500 mg initiation), he was able to communicate verbally, although with difficulties with phonation even with a Passy-Muir valve. There was orientation to person and place but not to time. His body temperature was 37.3 °C (99.1 °F) without antipyretic agents, while his WBC remained elevated at 20.9 (10^3^/uL) showing persistence of infection.

The finding of a neurological examination was consistent with critical illness polyneuropathy, as he had no motor function below the clavicle after surviving the profound neurological impairment induced by hyperpyrexia. His pain sensation remained intact, he responded to sharp and dull touch sensations, and he responded to vibrations. He was noted to have decreased cognitive speed; therefore, he required neurocognitive stimulation. Amantadine was initiated on day 34, resulting in improved cognitive functioning with noted increased cognitive speed during conversation. Amantadine was increased to 100 mg/ml via a PEG tube for enhancement of neuroprotection on day 41.

He was weaned off a ventilator, then transferred to our medical floor and eventually discharged. Extensive rehabilitation was recommended for neuromusculoskeletal strengthening in all his extremities. A CT scan showed diffuse brain atrophy disproportionate to his chronological age; thus consistent with hypoxic ischemic brain injury.

Following cognitive recovery, he was awake, alert, and oriented to time, place, person, and situation. He remained with a motor strength of 0/5 bilaterally in upper and lower extremities proximally and distally, secondary to critical illness polyneuropathy; however, he had intact temperature and pain sensation to all modalities in the upper and lower extremities, chest, abdomen, and face. He was seen on follow-up evaluation 6 months following initial encounter and he remained cognitively intact.

## Discussion

Body temperatures can be classified into different stages: hypothermia <35 °C (<95 °F), normal 36.5 to 37.5 °C (97.7 to 99.5 °F), fever 37.5 to 38.3 °C (99.5 to 100.9 °F), and hyperpyrexia 38.0 to 41.5 °C (100.4 to 106.7 °F) [2]. Of interest, neuronal damage may occur at the range of hyperthermia [12]. Significant in this case is an appreciable T_max_ of 41.6 °C (106.9 °F) followed by further neurocognitive decline from GCS 6 to GCS 3 within hours of increased core body temperature leading to coma and a persistent vegetative state, which posed a very poor recovery prognosis. In the event of hyperpyrexia, endoplasmic reticular stress occurs due to denaturing of the inherent polypeptide chains, nuclei carrying genetic information, and mitochondria [12]. In burn injuries, there is rapid destruction of polypeptides; however, in hyperpyrexia, as narrated in this case for instance, temperature raises gradually causing gradual dysfunction in the neuronal polypeptide chains [12]. One of the most evident indications of intracranial neuronal interference as noted in this case is decline in mental status and coma, which was supported by an EEG finding consistent with encephalopathy.

The OVLT is a brain structure present in the anteroventral third ventricle, which is connected to the median preoptic nucleus in the hypothalamus, which helps in regulating temperature, thirst, and vasopressin secretion [13]. The OVLT utilizes glutamate in creating neuronal excitation and action potential required in initiating temperature increase [8, 14]. Glutamate levels are known to increase slightly prior to a rise in body temperature during infectious processes [9]. Studies performed on rabbits showed evidence of OVLT glutamate spikes when staphylococcal enterotoxin A was injected [9]. In our case, while there was physiologic fever response given the presence of multiple infections, hyperpyrexia ensued thus damaging neuronal functioning as evidenced by the further decrease in GCS from 6 to 3 following a documented temperature of 41.6 °C (106.9 °F). Excess availability of glutamate in a brain with neuronal necrosis causes temperature regulation impairment thus mimicking a neurogenic fever which is difficult to regulate [14].

As observed in this case, following the noted rise in core body temperature, there was a decline in cognitive function predisposing our patient to further irreversible cognitive dysfunction similar to what is seen in neurogenic fever. Given that there were multiple sources of identified infections, which were poorly manageable using antibiotics, it was necessary to protect cognitive function by means of decreasing the amount of glutamate that was being produced in his brain. Werner *et al*. [15] documented that glutamate’s excitotoxicity mediated by the α-amino-3-hydroxy-5-methyl-4-isoxazolepropionic acid (AMPA)/kainate-type of glutamate receptors is known to cause damage to the neurons and the oligodendrocytes in the central nervous system. In this case, a similar scenario is proposed based on the finding of changes in mental status. The choice of levetiracetam was made for the purpose of decreasing the excitogenic effects of glutamate; it was found to be effective within 48 hours of administration as our patient became awake and alert following a prior comatose state for 21 days.

**Fig. 1:**
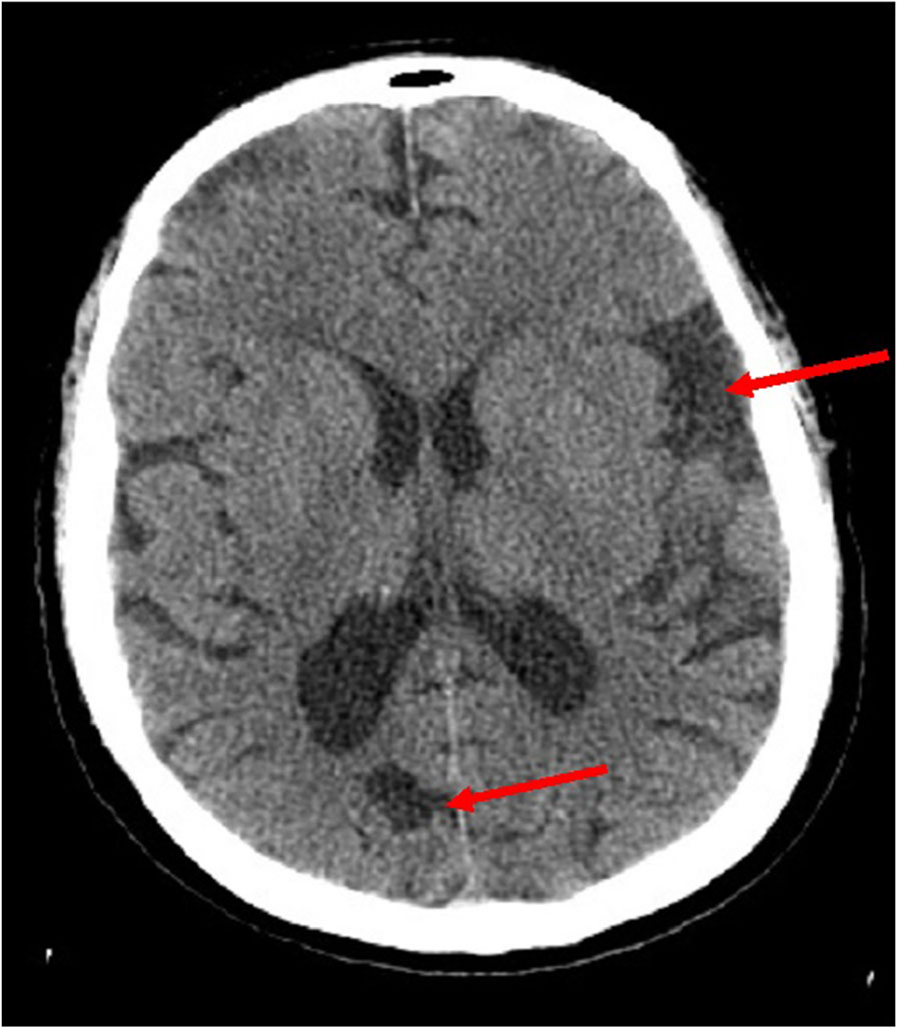
Computed tomography scan of brain without contrast showing brain atrophy disproportionately advanced for patient’s age. *Arrows* pointing to areas of atrophy

As an antiepileptic agent, levetiracetam functions by binding to neuronal synaptic vesicle glycoprotein 2A and inhibiting presynaptic calcium channels which leads to decreased cell excitation [11]. Prolonged excitatory states with glutamate release from brain neurons occur in anoxic-hypoxic brain injury via unregulated release of neurotransmitters from the vesicles where they are normally stored [16]. In hyperpyrexia, the excitatory states with continuous release of neurotransmitters further induce necrosis which usually can be noticed on CT imaging within 14 days of initial injury [17, 18] (Fig. 1). As further necrosis occurs, more glutamate is released from damaged neurons causing more excitatory states in the brain causing further cognitive decline and coma due to excitatory neuronal states. Excitogenicity caused by the release of glutamate after any form of brain injury occurs due to reactive oxygen stress, which occasionally causes epileptogenic foci in patients [19, 20]. In this case, our patient had hypoxic ischemic brain injury and brain atrophy as seen on CT imaging. Levetiracetam inhibits glutamate transmission through presynaptic P/Q-type calcium channels thus decreasing neuronal excitation [21]. The inhibition of glutamate release in our patient’s brain led to improvement and preservation in brain function by decreasing excitatory states in his brain and assisting in improved fever regulation. Hence, we theorize that the inhibition of action potential via utilization of levetiracetam halted the progression of neuronal damage in this patient thus preserving cognitive function despite a temperature of 41.6 °C (106.9 °F).

**Fig. 2:**
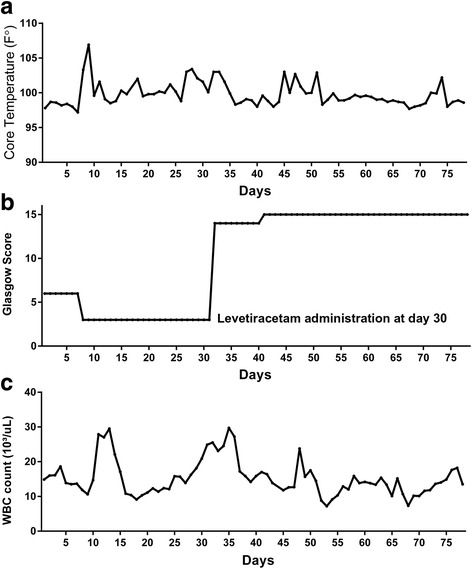
**a** Changes in core temperature. **b** Glasgow Coma Score. **c** Immune response before and after levetiracetam administration. *WBC* white blood cells

In the absence of pharmacological intervention with levetiracetam, a comatose state would have continued with persistent vegetative state given the excitatory state caused by glutamate release from damaged neurons; his comatose state would have become irreversible without intervention. As shown by his positive response to the regimen with levetiracetam within 48 hours of treatment, there is evidence to support that the treatment response in this patient was due to our pharmacological intervention. Without the prescribed regimental intervention, he had remained in a vegetative state for 21 days. However, within 9 days of treatment with levetiracetam, his cognitive function improved to the point that he was out of a comatose state and able to verbalize complaints (Fig. 2). Sepsis remained despite the cognitive intervention; leukocytosis persisted due to septic emboli from endocarditis requiring aortic valve replacement which was not performed during admission. He remained on antibiotic treatment even after discharge from our hospital and remained with persistent leukocytosis (Table 1).

**Table 1:** Choice of antibiotics based on antibiotics sensitivity studies

Day	WBC count (Range)	Temperature maximum	Antibiotic regimen based on culture and sensitivity studies	Microorganism
1–7	11.9–18.6	37.1 °C (98.7 °F)	Piperacillin-tazobactam 3.375 g intravenously Q 8 hours. Vancomycin 1 g intravenously Q 12 hours	*K. pneumoniae* – sputum
8–13	10.6–29.5	41.6 °C (106.9 °F)	Levofloxacin 500 mg intravenously Q 48 hours. Micafungin 100 mg intravenously Q daily	*K. pneumoniae* – sputum. *C. albicans* – blood
14–22	9.1–22.1	38.9 °C (102 °F)	Levofloxacin 500 mg intravenously Q 48 hours	*C. albicans* – blood and sputum
23–30	12.1–20.9	39.7 °C (103.4 °F)	Fluconazole 100 mg/50 ml, 100 mg Q 24 hours	*C. albicans* – blood
31–41	13.9–29.7	36.7–39.4 °C (98–103 °F)	Levofloxacin 500 mg intravenously Q 48 hours. Piperacillin-tazobactam 3.375 g intravenously Q 8 hours	*P. aeruginosa* – stool and wound
42–49	12.6–17.5	36.7–39.4 °C (98–103 °F)	Daptomycin 500 mg intravenously Q 8 hours	*S. epidermidis* – skin, stool, blood
50–57	7.2–17.5	36.8–39.4 °C (98.3–102.9 °F)	Metronidazole 500 mg intravenously Q 8 hours. Linezolid 600 mg/300 ml Q 12 hours	MRSA – sputum. *C. difficile* – stool
58–67	10.1–15.9	36.5–37.6 °C (97.7–99.7 °F)	Levofloxacin 750 mg intravenously Q 24 hours. Daptomycin 500 mg intravenously Q 8 hours	MRSA – sputum
68–78	7.3–18.2	36.5–39.0 °C (97.7–102.2 °F)	Vancomycin 1 g intravenously Q 12 hours. Imipenem 500 mg intravenously Q 8 hours	MRSA – Blood

C. albicans *Candida albicans*, C. difficile *Clostridium difficile*, K. pneumonia *Klebsiella pneumoniae*, *MRSA* methicillin-resistant *Staphylococcus aureus*, P. aeruginosa *Pseudomonas aeruginosa*, *Q* every, S. epidermidis *Staphylococcus epidermidis*, *WBC* white blood cells

The role of amantadine in this case was that of neurocognitive stimulation via dopamine agonist effects. In addition, amantadine has neuroprotective properties via inhibition of glutamatergic N-methyl-D-aspartate (NMDA) receptor reducing the release of proinflammatory factors from activated microglia and increasing the expression of glial cell line-derived neurotrophic factor (GNDF) in astroglia. Wakefulness, a function associated with dopamine, was further improved with the use of amantadine in this patient [22, 23].

Prior to treatment with amantadine, our patient was noted to have increased latency of response to questions following recovery from comatose state. Following initiation of amantadine 50 mg/mL via PEG tube daily on day 34 and 100 mg/mL via PEG tube daily on day 41, there was improvement in his cognitive speed as measured by improvement in speed of response, ability to initiate conversations, and spontaneous report of complaints. His GCS was 15 following the combined pharmacological intervention with levetiracetam and amantadine.

### Limitations

Our inability to obtain a magnetic resonance imaging (MRI) of his brain due to unavailability at our facility limits our evaluation of the structures of his brain following recovery from a coma. CSF analyses were not able to be obtained because he was on broad spectrum antibiotics and it was not deemed medically necessary at the time.

## Conclusions

Physicians should be aware that the combination of hyperpyrexia and cognitive changes including coma cause poor recovery due to neuronal brain damage. The utilization of levetiracetam and amantadine may provide neuroprotective and neurorestorative effects following hyperpyrexia with coexisting cognitive decline. Our case depicts the successful use of both medications in restoring cognitive function following hyperpyrexia with extensive comatose state. Given the successful recovery achieved in this case, we suggest that further research, such as randomized controlled trials, need to be explored in similar cases to harness the benefits of the conceptualization depicted in this case report.

## Abbreviations

CSF: Cerebrospinal fluid
CT: Computed tomography
EEG: Electroencephalogram
EGD: Esophagogastroduodenoscopy
GABA: Gamma-aminobutyric acid
GCS: Glasgow Coma Score
GNDF: Glial cell line-derived neurotrophic factor
MRI: Magnetic resonance imaging
MRSA: Methicillin-resistant *Staphylococcus aureus*
NMDA: N-methyl-D-aspartate
OVLT: Organum vasculosum laminae terminalis
PEG: Percutaneous endoscopic gastrostomy
TCA: Tricarboxylic acid
T_max_: Maximum core temperature
WBC: White blood cell
